# Fingerprint analysis of processed Rhizoma Chuanxiong by high-performance liquid chromatography coupled with diode array detection

**DOI:** 10.1186/s13020-015-0031-3

**Published:** 2015-02-10

**Authors:** Jia-Yan Fang, Lin Zhu, Tao Yi, Jian-Ye Zhang, Ling Yi, Zhi-Tao Liang, Li Xia, Jia-Fu Feng, Jun Xu, Yi-Na Tang, Zhong-Zhen Zhao, Hu-Biao Chen

**Affiliations:** School of Chinese Medicine, Hong Kong Baptist University, Hong Kong Special Administrative Region, Hong Kong, People’s Republic of China; School of Pharmaceutical Sciences, Guangzhou Medical University, Guangzhou, 510182 People’s Republic of China; School of Traditional Chinese Medicine, Guangdong Food and Drug Vocational College, Guangzhou, 510520 People’s Republic of China; Leshan Pharmaceutical Research Center, Leshan Vocational & Technical College, Leshan, 614000 People’s Republic of China

**Keywords:** Rhizoma Chuanxiong, HPLC-DAD, *Ligusticum chuanxiong*, Fingerprint, Processing

## Abstract

**Background:**

Rhizoma Chuanxiong (RC) is the dried rhizome of *Ligusticum chuanxiong* Hort., and various types of processed Rhizoma Chuanxiong (PRC) are widely used in China. However, quality assurance and quality control of these processed medicines remain challenging. This study aims to investigate the chemical compositions of various PRC preparations by a high-performance liquid chromatography (HPLC) coupled with diode array detection (DAD) method.

**Methods:**

A HPLC-DAD method with validation was developed for PRC samples. Seven batches of plant samples from two processing methods, stir-frying and steaming, were analyzed by the HPLC-DAD method. Common peaks in PRC chromatograms were chosen to calculate their relative retention time (RRT) and relative peak area (RPA), and similarity analyses of the chromatographic fingerprints were conducted by Similarity Evaluation System for Chromatographic Fingerprint of Traditional Chinese Medicine software (Version 2004 A).

**Results:**

In the 24-h stability test, the relative standard deviation for the RRT and RPA was less than 0.07% and 2.57%, respectively. The precision was less than 0.08% for the RRT and 2.48% for the RPA. The repeatability for the RRT and RPA was less than 0.03% and 2.64%, respectively. The similarities between the seven PRC batches were range from 0.956 to 0.990. After stir-frying or steaming, the amount of ferulic acid in PRC was much higher than that in the raw material.

**Conclusions:**

The fingerprint analysis of PRC by different processing methods was feasible by HPLC-DAD.

## Background

Many Chinese herbal medicines should be processed before clinical use, for achieving a unique function, reducing toxicity, enhancing efficacy, or stabilizing active ingredients [1,2]. Such processing involves many kinds of adjuvants. Rice wine and vinegar are the most widely used adjuvants [2]. Ginger juice, bran, and salt, are also often used [3,4]. Different processed products derived from the same botanic source are often used as the same herb. Hence, monitoring the quality of these processed medicines from different processing methods is critical [5].

Rhizoma Chuanxiong (RC), the dried rhizome of *Ligusticum chuanxiong* Hort. (Umbelliferae), is a major cardiovascular protective Chinese herb [6,7], especially for treating angina pectoris, cardiac arrhythmias, hypertension, and stroke [8,9]. RC also exhibits neuroprotective, anti-fibrotic, anti-nociceptive, anti-inflammatory, and anti-neoplastic activities [10-12]. Alkyl phthalides, phthalide dimers, and phenolic constituents were reported to be the main compounds responsible for the bioactivities and properties of RC [12-17]. Furthermore, some of the bioactive components were considered as drug candidates [18,19]. Ferulic acid, as a chemical marker of RC according to the Chinese Pharmacopoeia, is a main bioactive constituent [20]. Through our recent market investigation on RC, we found that stir-frying and steaming are the main processing methods, and that rice wine and vinegar are always used in the processing of RC.

Current analytical and quality control methods for RC simply focus on the detection of a few compounds by high-performance liquid chromatography (HPLC) and capillary electrophoresis [21,22]. Fingerprint analysis based on chromatography has been widely used for authentication and quality control of herbal drugs [23-26]. The combined HPLC and diode array detection (DAD) can provide online spectral information for each peak in a chromatogram, which has become a powerful tool for the rapid identification of the constituents in herbal products. Therefore, this study aims to investigate the chemical compositions of various processed RC (PRC) preparations by a HPLC coupled with DAD method.

## Methods

### Materials

Dried RC, which was produced in Sichuan, was purchased from Qingping Market of Guangzhou in mainland China. The authentication of the herbs was confirmed by Dr. Yi Tao according to the morphological features [6]. The dried rhizomes were sliced, and a total of seven batches of RC were obtained. Each batch had triplicate samples weighing 50 g. The seven batches of the herb were processed under different processing conditions (Table 1) [27].

**Table 1:** **Seven processing methods for PRC (** ***n*** **= 3)**

**Samples**	**Processing methods**	Additive and ratio (w:w)*	**Processing time (h)**
PRC-0	–	–	–
PRC-1	Stir-frying	–	0.5
PRC-2	Steaming	–	0.5
PRC-3	Stir-frying with rice wine	Rice wine (1:10)	0.5
PRC-4	Steaming with rice wine	Rice wine (1:10)	0.5
PRC-5	Stir-frying with vinegar	Vinegar (1:5)	0.5
PRC-6	Steaming with vinegar	Vinegar (1:5)	0.5

*The (w:w) values are the weight ratios of the additive to the herb.

### Reagents and chemicals

Rice wine was purchased from the Pagoda Brand (Zhejiang Pagoda Brand; Shaoxing Rice Wine Co. Ltd., China; alcohol: 15%) and vinegar was purchased from the Amoy Brand (Amoy Food Ltd., Hong Kong; acetic acid: 5–8%).

**Figure 1:**
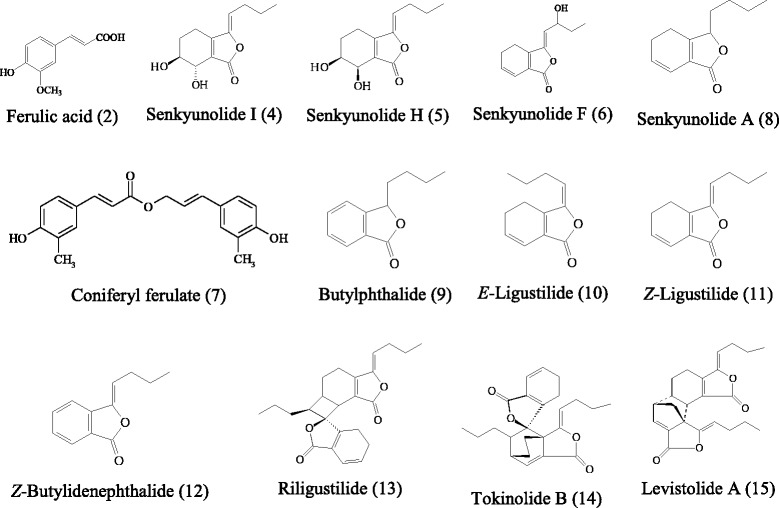
**Structures of the identified compounds in the fingerprints of the PRC samples.**

Ferulic acid was purchased from the National Institute for the Control of Pharmaceutical and Biological Products (China). The standard compounds of senkyunolide I, senkyunolide H, senkyunolide A, *Z*-ligustilide, and levistolide A were isolated from RC in our laboratory [28], and their structures were shown in Figure 1.

The acetonitrile and formic acid used in the HPLC analysis were of HPLC grade and obtained from Lab-scan (Thailand). The methanol used for the sample extraction was of analysis grade and also purchased from Lab-scan (Thailand). Deionized water was generated using a Milli-Q water purification system (Millipore, USA).

### HPLC- DAD instrumentation and conditions

HPLC-DAD was carried out by an Agilent 1100 series HPLC-DAD system comprising a vacuum degasser, binary pump, autosampler, thermostated column compartment, and DAD (Agilent, USA), which was used for acquiring chromatograms and UV spectra. An Alltima C_18_ column (5 μm; 4.6 × 250 mm) was used for HPLC analysis. The mobile phase consisted of 0.1% formic acid in water (A) and acetonitrile (B), and the procedure was performed with a gradient program of 15–20% (B) at 0–10 min, 20–53% (B) at 10–40 min, and 53–100% (B) at 40–60 min. The flow rate was 1 mL/min. The detection wavelength was set at 280 nm. The column temperature was set at 30°C. The injection volume was 10.0 μL.

### Preparation of standard and sample solutions

Standard solutions were prepared at a concentration of 50 mg/L with methanol. The seven batches of PRC were crushed with a grinder. The ground powder was passed through a 20-mesh (0.85-mm) sieve and stored at about 4°C before use. PRC powder (0.25 g) was immersed in 8 mL of methanol and ultrasonically extracted for 30 min at room temperature, and this procedure was repeated three times. The mixture was centrifuged (Eppendorf 5810 R, Hamburg, Germany) at 3200 × *g* for 10 min. The supernatant solution was transferred into a 25-mL volumetric flask, made up to full volume with methanol, and filtered through a nylon syringe filter (0.20 μm, Filtrex, USA) before HPLC analysis.

### Assay validation and sample determination

The method precision was determined by injecting the same extract of the PRC-0 sample six times in one day. The method repeatability was evaluated by analyzing six independently-extracted PRC-0 samples. The sample stability was assessed by the same PRC-0 extract at 0, 2, 4, 8, 16, and 24 h. The similarity evaluation of PRC was performed on seven batches of processed samples.

The data analysis was conducted by Similarity Evaluation System for Chromatographic Fingerprint of Traditional Chinese Medicine software (Version 2004 A), which was recommended by the State Food and Drug Administration of China. The software was used to calculate the correlation coefficients of the chromatographic profiles of the seven batches of PRC samples, and to generate a simulative mean chromatogram (SMC). The similarities of different chromatographic fingerprints were compared by the PRC-0 samples.

## Results and discussion

### Identification of major constituents in various PRC samples

**Figure 2:**
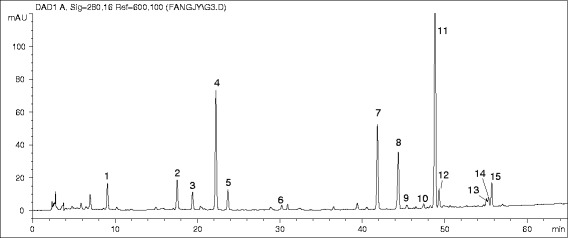
**Typical chromatogram of the PRC-0 sample at 280 nm.**

Typical chromatogram of the PRC was shown in Figure 2. Based on comparisons with standard compounds, six peaks were identified as feruled acid (2), senkyunolide I (4), senkyunolide H (5), senkyunolide A (8), *Z-*ligustilide (11), and levistolide A (15). Seven other peaks were tentatively identified as senkyunolide F (6), coniferyl ferulate (7), butylphthalide (9), *E-*ligustilide (10), *Z-*butylidenephthalide (12), riligustilide (13), and tokinolide B (14) by comparing their retention times and UV spectra with the standard compounds. From the assay results, the major constituents in the various PRC samples were similar, namely phenolic constituents, alkylphthalides, and phthalide dimers.

### Validation of the fingerprint analysis method

The fingerprints of the PRC samples were obtained by HPLC in 60 min. Fifteen common peaks were found in the HPLC fingerprint chromatograms of the seven batches of PRC samples. Peak 7 (coniferyl ferulate) was assigned as the reference peak to conduct method validation, because it had a moderate retention time and peak area compared with the other peaks. The HPLC method was validated in terms of stability, precision, and repeatability, and the results were listed in Table 2. In the 24-h stability test, the relative standard deviation (RSD) of the relative retention time (RRT), defined as the ratio of the retention time of the individual peak to that of the reference peak, and the relative peak area (RPA), defined as the ratio of the retention peak area of the individual peak to that of the reference peak, was less than 0.07% and 2.57%, respectively. The precision was less than 0.08% for the RRT and 2.48% for the RPA. The repeatability for the RRT and RPA was less than 0.03% and 2.64%, respectively. These data indicated that the HPLC method was suitable for fingerprint analysis of various PRC samples.

**Table 2:** **Validation of the fingerprint method (** ***n*** **= 3)**

	**Stability (RSD, %)**		**Precision (RSD, %)**		**Repeatability (RSD, %)**	
**Peak No.**	**RRT**	**RPA**	**RRT**	**RPA**	**RRT**	**RPA**
1	0.04	2.06	0.06	2.07	0.03	0.67
2	0.06	2.57	0.07	2.40	0.02	1.14
3	0.07	2.57	0.08	2.25	0.02	1.55
4	0.05	1.98	0.06	1.79	0.03	0.66
5	0.05	1.96	0.05	1.77	0.03	0.83
6	0.03	1.81	0.04	2.05	0.01	1.09
7(S)	—	—	—	—	—	—
8	0.01	1.89	0.00	1.69	0.01	0.69
9	0.01	1.80	0.00	2.48	0.01	2.32
10	0.01	2.45	0.01	2.31	0.01	2.20
11	0.01	1.93	0.01	1.73	0.02	0.46
12	0.01	2.15	0.01	2.44	0.02	2.19
13	0.02	1.33	0.01	0.65	0.03	1.77
14	0.02	1.26	0.01	1.32	0.03	2.64
15	0.02	1.60	0.02	2.04	0.03	1.72

### Fingerprint analysis of various PRC samples

Seven batches of PRC samples were analyzed with the present method. Using the Similarity Evaluation System for Chromatographic Fingerprint of Traditional Chinese Medicine (Version 2004 A), the RRT and RPA of the 15 common fingerprint peaks were calculated, and the results are shown in Table 3 with their mean and RSD values, respectively. The RSD values for the RRT fell in the range of 0.01–0.76%, while the RSD values of the RPA were observed in the range of 52.1–75.7%. The RSD values for the RRT were less than 0.76%, indicating that the fingerprint analysis was precise. The RSD values for the RPA were much larger, demonstrating that the processing methods had affected the contents of the compounds in the PRC samples. Although the contents varied, this fingerprint analysis by HPLC was still feasible and repeatable.

**Table 3:** **RRT and RPA of common peaks in seven batches of PRC (** ***n*** **= 3)**

**Peak No.**	**PRC-0**		**PRC-1**		**PRC-2**		**PRC-3**		**PRC-4**		**PRC-5**		**PRC-6**		**Average**		**RSD (%)**	
	**RRT**	**RPA**	**RRT**	**RPA**	**RRT**	**RPA**	**RRT**	**RPA**	**RRT**	**RPA**	**RRT**	**RPA**	**RRT**	**RPA**	**RRT**	**RPA**	**RRT**	**RPA**
1	0.22	0.20	0.21	0.27	0.21	0.69	0.21	0.30	0.22	1.46	0.21	0.46	0.21	0.63	0.21	0.57	0.39	75.7
2	0.42	0.28	0.42	0.68	0.42	1.30	0.42	0.55	0.42	2.97	0.42	1.48	0.42	1.11	0.42	1.20	0.05	74.6
3	0.46	0.16	0.46	0.23	0.46	0.34	0.46	0.20	0.46	0.68	0.46	0.57	0.46	0.42	0.46	0.37	0.76	53.2
4	0.53	1.07	0.53	2.00	0.53	3.10	0.53	1.83	0.53	6.64	0.53	3.78	0.53	2.73	0.53	3.02	0.04	60.4
5	0.57	0.18	0.57	0.34	0.57	0.52	0.57	0.30	0.57	1.13	0.57	0.62	0.57	0.47	0.57	0.51	0.03	61.1
6	0.72	0.05	0.72	0.08	0.72	0.12	0.72	0.07	0.72	0.25	0.72	0.14	0.72	0.11	0.72	0.12	0.02	55.2
7(S)	1	1	1	1	1	1	1	1	1	1	1	1	1	1	1	1	–	–
8	1.06	0.70	1.06	1.14	1.06	1.95	1.06	1.01	1.06	3.31	1.06	1.78	1.06	1.68	1.06	1.65	0.01	52.1
9	1.09	0.05	1.09	0.07	1.09	0.11	1.09	0.06	1.09	0.23	1.09	0.13	1.09	0.10	1.09	0.11	0.03	57.6
10	1.13	0.06	1.13	0.09	1.13	0.14	1.13	0.07	1.13	0.26	1.13	0.16	1.13	0.12	1.13	0.13	0.02	53.4
11	1.17	3.01	1.17	3.73	1.17	6.81	1.17	3.76	1.17	13.9	1.17	6.50	1.17	6.23	1.17	6.28	0.02	58.9
12	1.18	0.12	1.18	0.16	1.18	0.25	1.18	0.15	1.18	0.57	1.18	0.29	1.18	0.23	1.18	0.25	0.02	60.0
13	1.31	0.02	1.32	0.12	1.32	0.17	1.32	0.10	1.32	0.36	1.32	0.24	1.32	0.16	1.32	0.17	0.22	65.1
14	1.32	0.08	1.32	0.13	1.32	0.23	1.32	0.12	1.32	0.47	1.32	0.27	1.32	0.20	1.32	0.21	0.19	61.0
15	1.33	0.23	1.33	0.33	1.33	0.51	1.33	0.29	1.33	1.03	1.33	0.65	1.33	0.44	1.33	0.50	0.04	55.4

**Figure 3:**
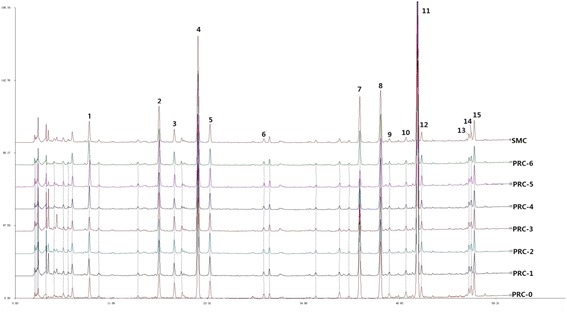
**HPLC-DAD fingerprints of seven batches of PRC and SMC at 280 nm.**

The chromatograms of the PRC samples from the seven processing methods and the SMC were shown in Figure 3. The results of the similarity analysis were listed in Table 4. The similarities between the seven batches of PRC samples were found to range from 0.956 to 0.990 (with PRC-0 serving as a reference), and the influences of processing on RC were as follows: PRC-5 (stir-frying with vinegar) > PRC-4 (steaming with wine) > PRC-2 (steaming) > PRC-1 (stir-frying) > PRC-6 (steaming with vinegar) > PRC-3 (stir-frying with wine) > PRC-0 (raw material). From these results, the processing methods of stir-frying with vinegar and steaming with wine caused significant differences in the chemical composition of RC after processing. From Table 3, the RPA values of the 15 constituents (with peak 7 assigned as a reference) in the various PRC samples all increased, compared with those in the raw material, indicating that the amounts of the 15 compounds in RC increased after processing.

**Table 4:** **Similarities of seven batches of PRC**

**Batch**	**Similarity**
PRC-0	1.000
PRC-1	0.980
PRC-2	0.978
PRC-3	0.990
PRC-4	0.963
PRC-5	0.956
PRC-6	0.982

As shown in Figure 3, the chromatogram revealed that peak 7 (coniferyl ferulate) was one of the major compounds in raw RC. Coniferyl ferulate was found to be susceptible to hydrolysis into ferulic acid in neutral, strongly acidic, and basic solvents, where heat and water could facilitate the hydrolysis [29]. This could result in the variable amounts of ferulic acid determined. In the present study, the ratios of ferulic acid to coniferyl ferulate in RC after processing with different methods were much higher than that in the raw material. This could also be explained by the increase in the amount of ferulic acid in RC after processing. Ferulic acid is known to be the active component of PRC and responsible for its therapeutic effects, and the enhancement of ferulic acid could increase its biological effects in herbs [1,6].

Many Chinese herbal medicines need to be processed before clinic use. However, the study on the quality assurance and quality control (QA & QC) of these processed medicines is still inadequate. In our manuscript, a fingerprint analysis by HPLC-DAD method was developed and well validated to evaluate PRC, a typical Chinese herbal medicine. Base on the present study, our result indicates HPLC-DAD fingerprinting is a powerful approach for QA & QC of PRC, which can be applied to other processed medicines. Thus, this study contributes to the quality study of Chinese medicines, especially for the research of processed Chinese medicine by HPLC-DAD fingerprinting.

## Conclusions

The fingerprint analysis of PRC by different processing methods were feasible by HPLC-DAD.

## Abbreviations

HPLC: High performance liquid chromatography
DAD: Diode array detection
PRC: Processed Rhizoma Chuanxiong
RRT: Relative retention time
RPA: Relative peak area
RSD: Relative standard deviation
QA: Quality assurance
QC: Quality control
